# The primitive growth factor NME7_AB_ induces mitochondrially active naïve-like pluripotent stem cells

**DOI:** 10.1016/j.bbrep.2019.100656

**Published:** 2019-08-20

**Authors:** Carla O'Reilly, Qian Qi, Jennifer L. Peters, Yong Cheng, Sang-Oh Yoon, Min-Joon Han

**Affiliations:** a Department of Hematology, St. Jude Children's Research Hospital, 262 Danny Thomas Place, Memphis, TN, 38105, United States; b Cell and Tissue Imaging Center, Light Microscopy Shared Resource, St. Jude Children's Research Hospital, 262 Danny Thomas Place Memphis, TN, 38105, United States; c University of Illinois College of Medicine, Peoria, IL, 61605, United States

**Keywords:** Naïve stem cell, Mitochondria, Oxidative phosphorylation, ERK/Smad signaling

## Abstract

Naïve pluripotent stem cells (PSCs) display a distinctive phenotype when compared to their “primed” counterparts, including, but not limited to, increased potency to differentiate and more robust mitochondrial respiration. The cultivation and maintenance of naïve PSCs have been notoriously challenging, requiring the use of complex cytokine cocktails. NME7_AB_ is a newly discovered embryonic stem cell growth factor that is expressed exclusively in the first few days of human blastocyst development. It has been previously reported that growing primed induced PSCs (iPSCs) in bFGF-depleted medium with NME7_AB_ as the only added growth factor facilitates the regression of these cells to their naïve state. Here, we confirm this regression by demonstrating the reactivation of mitochondrial function in the induced naïve-like PSCs and increased ATP production in these cells, as compared to that in primed iPSCs.

## Highlights

• NME7_AB_ treatment is sufficient for the regression of primed iPSC to naïve state stem cell.
• NME7_AB_ derived naïve state stem cell shows an active mitochondrial function.
• NME7AB derived naïve-like PSCs show ERK independent Smad2 activation.

## 1 Introduction

Pluripotent stem cells (PSCs) are distinguished from other cell types by several characteristics; the most prominent of these is their ability to divide without losing their capacity for long-term self-renewal, thereby maintaining the ability to differentiate based on external signaling. The PSC designation encompasses various subtypes that are present during different stages of development. In pre-implantation embryos, cells arise from the inner cell mass of the early blastocyst and are referred to as “naïve” PSCs. These cells give rise to “primed” PSCs during post-implantation development [1]. Naïve and primed PSCs differ with respect to their metabolism, growth-factor dependence, and X-chromosome inactivation [2].

Mitochondria are at the forefront of cellular metabolism and are ubiquitous and essential organelles that generate 90% of the adenosine triphosphate (ATP) needed for eukaryotic cellular function. Mitochondria also play a crucial role in regulating programmed cell death by mediating many cellular signaling pathways [3,4]. The mitochondrial matrix generates the major metabolites and intermediates of four biochemical pathways: the tricarboxylic acid (TCA) cycle, the urea cycle, oxidative phosphorylation (OXPHOS), and fatty acid oxidation [3]. Therefore, mitochondria play a vital role in metabolism through monitoring and managing energy production and expenditure. Recently published data have shown that not only cancer cells but also primed PSCs (e.g., embryonic stem cells [ESs] or induced pluripotent stem cells [iPSCs]) preferentially generate ATP through glycolysis in the cytoplasm (a phenomenon called the Warburg effect [5]) instead of through OXPHOS in the mitochondria [[6], [7], [8]]. Still other studies have suggested that, in contrast to primed PSCs, naïve PSCs retain active mitochondrial function [[9], [10], [11]].

NME7_AB_ is a newly discovered embryonic stem cell growth factor that is present in human blastocysts only during the first few days of development [12]. Evidence suggests that when stem cells are grown in culture in minimal medium containing NME7_AB_ as the only added growth factor, the primed PSCs revert to a state equivalent to that of pre-implantation naïve-like PSCs [12]. Later in embryonic development, NME7_AB_ is replaced by another NME family member, NME1, which limits pluripotency by changing its multimerization state from an active dimer to an inactive hexamer [13,14]. NME7_AB_ may support the early naïve state, whereas NME1 supports a subsequent intermediary naïve-like state in the transition to primed PSCs.

These findings led us to investigate the changes in mitochondrial function at different stages of PSC development. We converted primed PSCs to naïve-like PSCs by adding recombinant NME7_AB_. This is a simple, feeder-free, and chemically defined method. In addition, the naïve-like PSCs produced by this method exhibit full capacity for pluripotency and have a more active mitochondrial function when compared to primed PSCs.

## 2 Materials and methods

### 2.1 iPSC culture

Human iPSCs were generated by infecting human female dermal fibroblasts (PCS-201-012 cells, purchased from ATCC) with a Sendai virus vector encoding four reprogramming factors (L-MYC, OCT4, SOX2, and KLF4) (Invitrogen, Carlsbad, CA). Embryonic stem cell–like colonies were formed after 3 weeks of viral infection, and the observed ES-like colonies were manually picked and transferred to mouse feeder cells (MEFs) to generate iPSC lines. The iPSCs were maintained in ES medium (Dulbecco's Modified Eagle Medium Nutrient Mixture F12 (DMEM/F12; Invitrogen) supplemented with 2 mM l-glutamine (Invitrogen), 1 mM β-mercaptoethanol, 1 × nonessential amino acids (NEAA; Invitrogen), 20% knockout serum replacement (KOSR; Invitrogen), and 10 ng/mL basic fibroblast growth factor (bFGF; Invitrogen). Until an iPSC line was established, iPSC colonies were mechanically picked. The established iPSC cell lines were maintained in mTeSR Human Embryonic Stem Cell Culture Medium (STEMCELL Technologies, Vancouver, BC, Canada) and passaged by ReLeSR reagent (STEMCELL Technologies) under Geltrex™ LDEV-Free hESC-Qualified Reduced Growth Factor Basement Membrane Matrix (Invitrogen). Cells were maintained at 37 °C in a hypoxic incubator (5% CO_2_, 5% O_2_).

### 2.2 Naïve cell culture

Human naïve cells were maintained in NME7_AB_ medium (DMEM/F12; Invitrogen) supplemented with 2 mM l-glutamine (Invitrogen), 1 mM β-mercaptoethanol, 1 × nonessential amino acids (NEAA; Invitrogen), 20% knockout serum replacement (KOSR; Invitrogen), and NME7_AB_. The cells were plated in an MN-C3 antibody–coated plate with 10 μM Y-27632 Rho kinase I inhibitor and maintained at 37 °C in a hypoxic incubator (5% CO_2_, 5% O_2_).

### 2.3 Western blot analysis

Protein samples (50 μg) were electrophoresed on 12% SDS-PAGE gels and transferred to Immun-Blot NC membranes (Bio-Rad, Richmond, CA). Each membrane was blocked for 3–5 h in Tris-buffered saline (TBS) containing 0.1% Tween-20 and 5% (w/v) dry skim-milk powder and then incubated overnight at 4 °C with primary antibodies. The membrane was washed three times with TBS containing 0.05% Tween-20 (TBST) then incubated for 2 h with the appropriate secondary antibody (Pierce, Rockford, IL). After washing twice with TBST and once with TBS, the bound antibody was imaged with the Li-COR system. Antibodies to GAPDH, Smad2, phospho-Smad2, ERK1/2, and phospho-ERK1/2 were purchased from Cell Signaling Technology (Danvers, MA).

### 2.4 Immunofluorescence

Cells were fixed immediately with fixing solution (2% paraformaldehyde, 100 mM KCl, 200 mM sucrose, 1 mM EGTA, 1 mM MgCl_2_, 10 mM PIPES, pH 6.8) for 10 min and then treated with permeabilization buffer (0.2% Triton X-100, 100 mM KCl, 200 mM sucrose, 1 mM EGTA, 1 mM MgCl_2_, 10 mM PIPES, pH 6.8) for 10 min after washing with PBS. The cells were then washed three times with PBS and incubated for 15 min with blocking solution containing 3% BSA in PBS. The cells were then washed a further three times with PBS and incubated overnight with the primary antibodies in blocking solution. Antibodies to OCT4 and H3K27me3 were purchased from Cell Signaling Technology. Next day, the cells were washed three times with PBS and incubated with the secondary antibody, a goat anti-mouse antibody conjugated with Alexa Fluor® 488 fluorescent dye (Molecular Probes, Eugene, OR), in blocking solution. After further washing with PBS, the cells were stained with Hoechst dye to stain the nuclei. Each image was examined using an EVOS fluorescence microscope (Invitrogen).

### 2.5 Mitochondrial activity measurement

Mitochondrial activity was measured using a Seahorse XF24 analyzer (Agilent, Santa Clara, CA). The Seahorse XF24 analyzer allowed the simultaneous quantification of the mitochondrial respiration rate (the oxygen consumption rate [OCR]). Three mitochondrial inhibitors (1 μM oligomycin, 1 μM FCCP, and 0.5 μM rotenone/antimycin A; Agilent) were used in succession to monitor the mitochondrial activity in naïve and primed iPSCs.

### 2.6 Reverse-transcription polymerase chain reaction (RT-PCR)

Total RNA was extracted from cells by using a Direct-zol RNA Purification Kit (Zymo Research, Irvine, CA), and cDNA was synthesized with the ThermoScript™ RT-PCR System (Invitrogen). For quantitative analysis, Q-PCR was performed with SYBR green, using the following conditions: an initial denaturation at 95 °C for 3 min, followed by 40 cycles of amplification, with each cycle consisting of 95 °C for 10 s and 60 °C for 30 s. The following primers were used: for hOCT4, GCTCGAGAAGGATGTGGTCC and CGTTGTGCATAGTCGCTGCT; for hSOX2, AACCCCAAGATGCACAACTC and CGGGGCCGGTATTTATAATC; for hSOX17 (an endoderm marker), GAATCCAGACCTGCACAACG and CTCTGCCTCCTCCACGAAG; for hBRACHURY (a mesoderm marker), ACTCACCTGCATGTTTATCCA and CCGTTGCTCACAGACCACAG; and for hGFAP (an ectoderm marker), TGGAGGTTGAGAGGGACAAT and TAGGCAGCCAGGTTGTTCTC.

### 2.7 ATP determination assays

Cellular ATP concentrations were measured with an ATP Determination Kit (Molecular Probes). Cells were washed three times with PBS and boiled for 5 min in water to lyse them. Cell lysates were collected by centrifugation at 12000×*g* for 15 min at 4 °C. To measure the ATP, chemiluminescent detection was performed using firefly luciferase and luciferin, with the signal being measured by a SpectraMax Microplate Reader (Molecular Devices, San Jose, CA). The protein concentration of the cell lysate was determined by BCA assay (Bio-Rad), and the result in RLU (relative luminescent units) was normalized to the protein concentration.

### 2.8 Three-germ-layer differentiation

The naïve-like and primed iPSCs were plated on Geltrex-coated plates after undergoing single-cell dissociation. Three-germ-layer differentiation was performed by using a STEMdiff™ Trilineage Differentiation Kit (STEMCELL Technologies) according to the manufacturer's protocol. To validate the expression of each germ-layer differentiation, Q-PCR and immunofluorescence assays were performed with the following antibodies: anti-OTX2 (for ectoderm), anti-BRACHYURY (for mesoderm), and anti-SOX17 (for endoderm). All antibodies were purchased from R&D Systems.

### 2.9 RNA-seq

Total RNA was extracted using an RNeasy Plus Micro Kit (Qiagen). cDNA libraries were constructed using an Illumina TruSeq Stranded mRNA Kit with poly-A selection. Libraries were paired-end 100-bp sequenced using an Illumina HiSeq 2500 System. The sequencing reads were aligned to human cDNA from ensembl.org by using Kallisto [19] (version 0.43.0) with the default settings. Differentially expressed genes were called using the Sleuth R package [20].

### 2.10 Transmission electron microscopy

Samples were fixed overnight in 2.5% glutaraldehyde, 2% paraformaldehyde in 0.1 M sodium cacodylate buffer, pH 7, then post fixed for 1.5 h in 2% osmium tetroxide in 0.1 M cacodylate buffer with 0.3% potassium ferrocyanide. After the tissue was rinsed in the same buffer, it was stained with 4% aqueous uranyl acetate and dehydrated through a graded ethanol series to propylene oxide. It was then infiltrated through a propylene oxide:epon series, ending with 100% epon overnight. This routine processing was performed on a Leica EM TP Tissue Processor. Next day, the tissue was embedded in fresh epon and polymerized at 70 °C overnight. Semithin sections (0.5 μm) were stained with toluidine blue for light microscope examination. Ultrathin sections (80 nm) were cut and imaged using an FEI Tecnai 200Kv FEG Electron Microscope with an ATM XR41 2K Digital Camera.

## 3 Results and discussion

### 3.1 Generation of human iPSCs and conversion to naïve-like PSCs

**Fig. 1:**
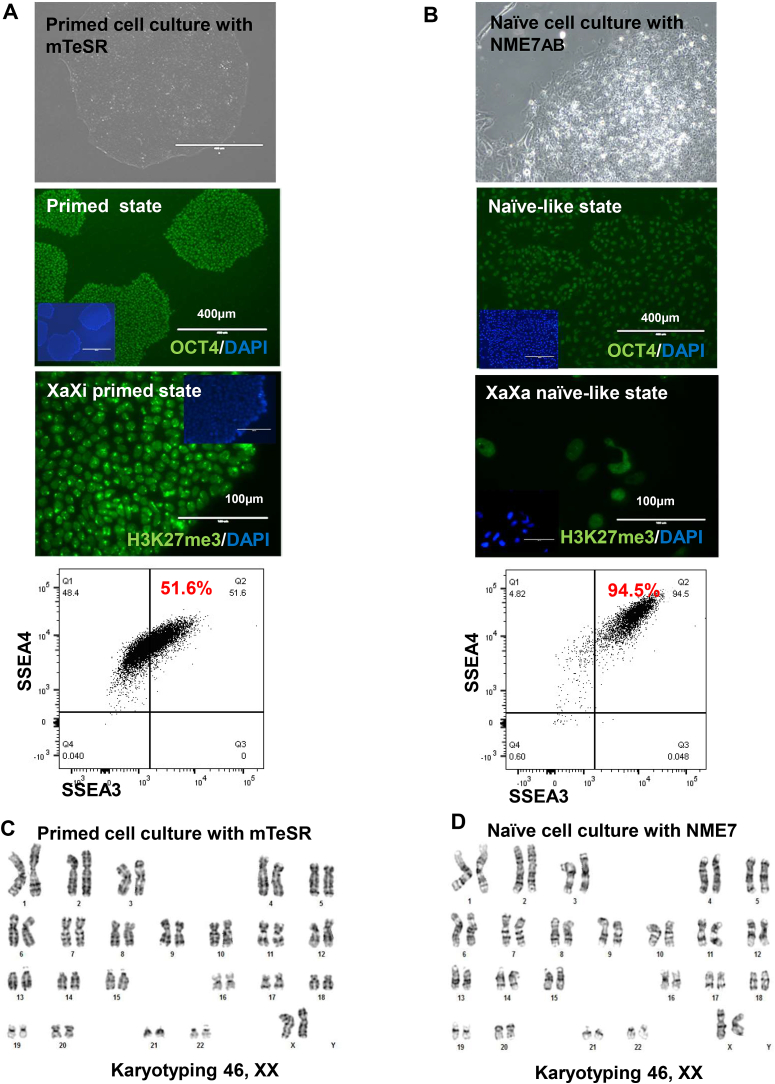
**Naïve-like stem cell conversion by adding NME7_AB_**. **(A)** Human female primed iPSC lines were generated by treating human female dermal fibroblast cells with four reprogramming factors (c-Myc, OCT4, SOX2, and KLF4) encoded by a Sendai virus vector. To confirm the stemness of the iPSCs, immunofluorescence assays were performed with an anti-OCT4 antibody (green). DAPI staining (blue) was used to show nuclei. The scale bar in this image represents 400 μm. Antibodies to SSEA-3 and SSEA-4 were used for flow analysis. Primed stem cells had one active X chromosome; the other X chromosome had been inactivated through methylation (indicated by condensed dot/punctate staining with the anti-H3K27me3 antibody; the scale bar in the image represents 100 μm). **(B)** After cells were converted to a naïve-like state by adding NME7_AB_, their stemness was confirmed by immunofluorescence assays performed with anti-OCT4 antibody (green). Hoechst staining (blue) was used to show nuclei. The scale bar in this image represents 400 μm. Antibodies to SSEA-3 and SSEA-4 were used for flow analysis. Naïve stem cells had two active X chromosomes (shown by cloud-like staining with an anti-H3K27me3 antibody; the scale bar in the image represents 100 μm). **(C)** The primed iPSC cells showed normal 46, XX karyotyping. **(D)** The naïve-like stem cells showed normal 46, XX karyotyping.

Human iPSC lines were generated by treating human female dermal fibroblast cells with a Sendai virus vector, which is an established non-integration method for reprogramming. Once the iPSC lines were established, the cells were cultivated under feeder-free conditions to prevent contamination by mouse feeder cells in downstream functional assays. Immunofluorescence assays with an antibody to the canonical pluripotency marker OCT4 and flow cytometry analysis with antibodies to SSEA3/SSEA4 confirmed the pluripotency of the established iPSCs (Fig. 1A). From among these established iPSC lines, single clonal cells that showed non-viral gene integration were used for the subsequent experiments. In an earlier study, primed human iPSCs were converted to a naïve state by growing them in culture in serum/bFGF-free medium containing a primitive growth factor, NME7_AB_ [12]. We also used NME7_AB_ to generate naïve-like stem cells, congruent with this previously published method. To verify the conversion, we used the H3K27 trimethylation (H3K27me3) marker. Primed iPSCs have one active and one inactive X chromosome, whereas naïve stem cells have two active X chromosomes. In primed iPSCs, staining with an anti-H3K27me3 antibody resulted in condensed puncta, signifying X-chromosome inactivation (Fig. 1A). In contrast, X-chromosome reactivation resulted in cloud-like staining with the anti-H3K27me3 antibody (Fig. 1B), and this can be seen after the conversion of primed PSCs to naïve-like (XaXa) PSCs. However, the resulting naïve-like stem cells still expressed the pluripotent markers OCT4 and SSEA3/SSEA4 at high levels (Fig. 1B). Both primed PSCs and naïve-like PSCs showed normal karyotyping (Fig. 1C and D).

**Fig. 2:**
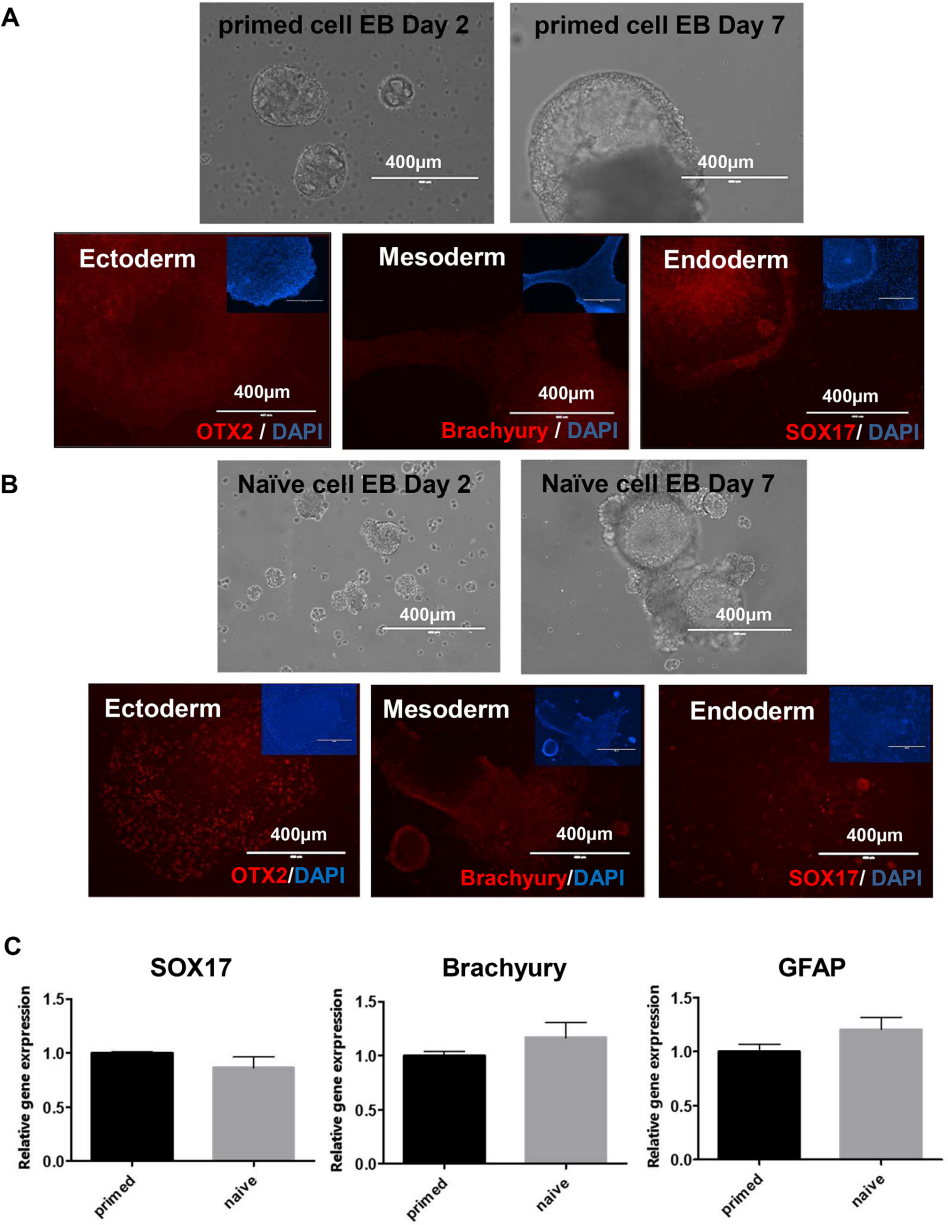
**Pluripotency of naïve-like stem cells converted by adding NME7_AB_**. **(A)** The primed iPSCs were differentiated into three germ layers. **(B)** Naïve-like PSCs were differentiated into three germ layers: ectoderm (expressing OTX2), mesoderm (expressing brachyury), and endoderm (expressing Sox17). Hoechst staining (blue) was used to show nuclei. The scale bars in all images in panels A and B represent 400 μm. **(C)** After three-germ-layer differentiation, the expression of each germ layer–specific gene level was determined by Q-PCR. The data are shown as the mean ± SD of triplicate experiments.

Naïve-like PSCs have full “potency of differentiation.” To explore this capability in primed and naïve-like PSCs, we applied several different differentiation techniques. Initially, primed and naïve-like PSCs were differentiated into embryoid bodies (EBs)—three-dimensional aggregates of PSCs—and supported pluripotency. Next, primed and naïve-like PSCs (Fig. 2A and B, respectively) were differentiated into three germ layers: ectoderm, mesoderm, and endoderm. After the differentiation of each lineage, the cells were stained with antibodies to specific lineage markers: OTX2, brachyury, and SOX17 (red) for ectoderm, mesoderm, and endoderm, respectively. Both primed and naïve-like PSCs could also be equally differentiated into the neuronal lineage and the hematopoietic lineage (Supplementary Fig. S1). For quantitative analysis, each set of germ layer–differentiated cells were analyzed by Q-PCR with primers to the genes encoding specific lineage markers: GFAP, Brachyury, and SOX17 for ectoderm, mesoderm, and endoderm, respectively (Fig. 2C).

### 3.2 The genetic signature of naïve-like PSCs and the corresponding signaling pathways

**Fig. 3:**
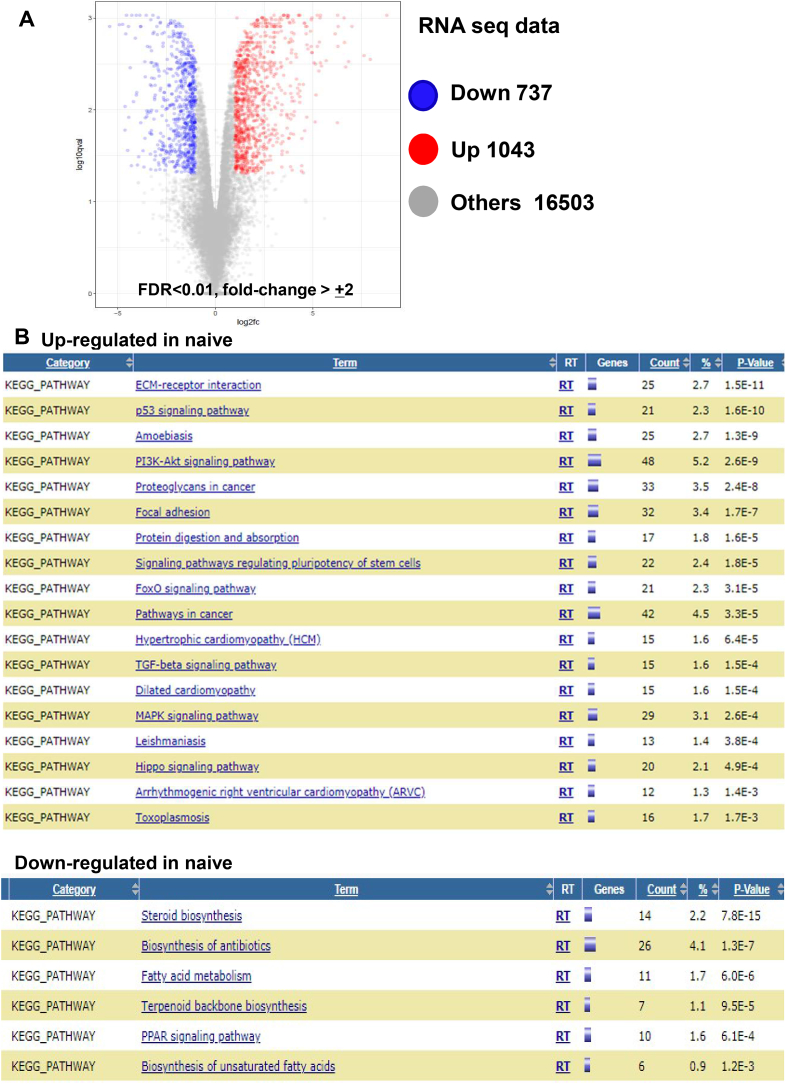
**Genetic signature of naïve-like stem cells converted by adding NME7_AB_**. **(A)** Genes that were differentially expressed in naïve-like and primed stem cells were identified by RNA-seq analysis. **(B)** KEGG pathway analysis was performed for the differentially expressed genes in naïve-like and primed stem cells.

**Fig. 4:**
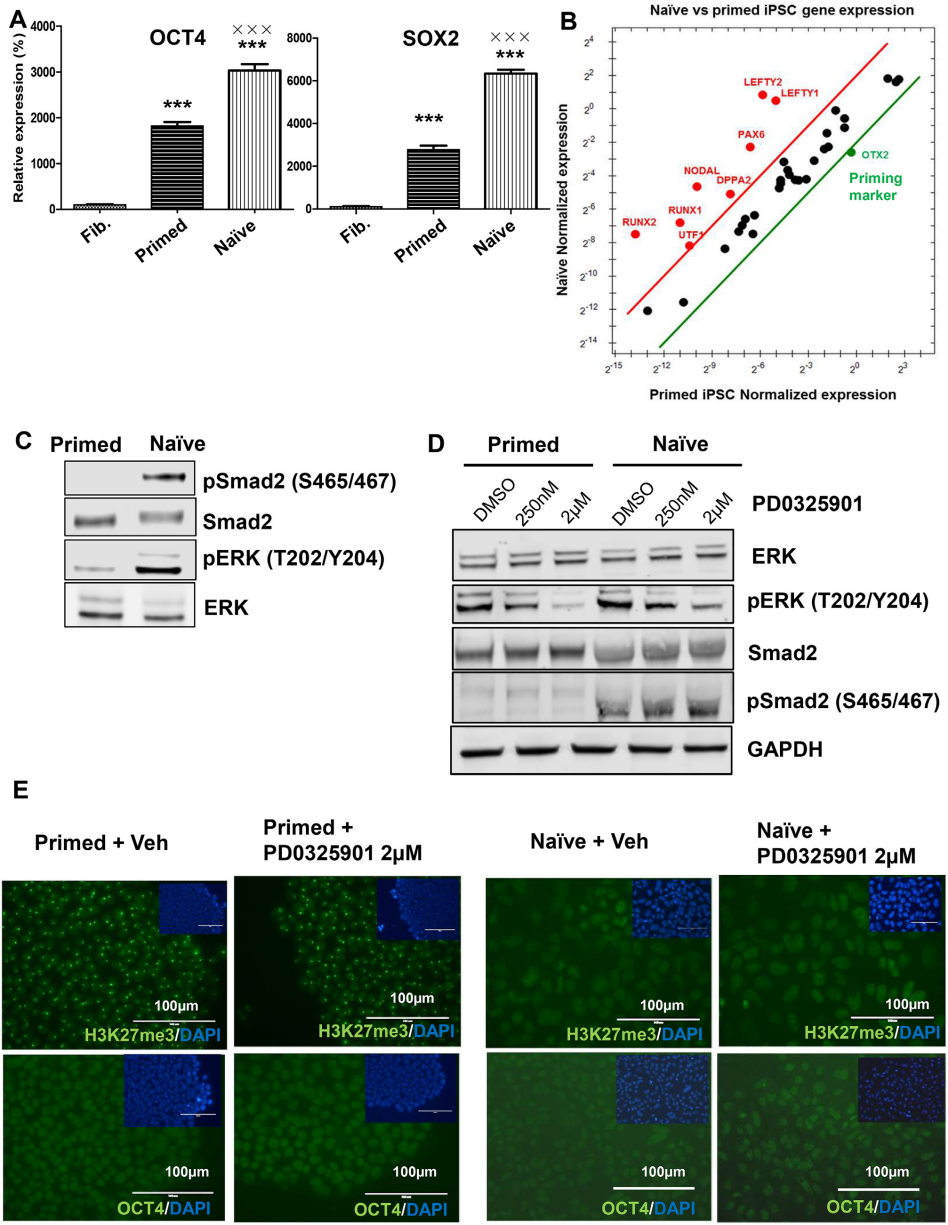
**ERK independent Smad2 activation in naïve-like stem cells converted by adding NME7_AB_**. **(A)** Naïve-like stem cells showed upregulation of pluripotent genes (i.e., *OCT4* and *SOX2*) when compared with the original fibroblast cells and primed PSCs. The data are shown as the mean ± SD of triplicate experiments. The statistical analysis was performed using GraphPad Prims. Statistical test among the different groups used 1-way ANOVA (***P < 0.01 compared to fibroblast cells, **×××***P* < 0.01 compared to primed cells). **(B)** Naïve-like stem cells showed upregulation of pluripotent stem cell markers and downregulation of the priming marker OTX2. **(C)** The naïve-like stem cells showed hyperactivation of ERK and Smad2 signaling, as measured using anti-Smad2/phopho-Smad2 and anti-ERK/phosphor-ERK antibodies. **(D)** Naïve-like and primed stem cells grown in the presence of the MEK inhibitor PD0325901 were lysed, and the activities of Smad2 and ERK1/2 were measured using anti-Smad2/phopho-Smad2 and anti-ERK/phosphor-ERK antibodies, respectively. **(E)** Naïve-like and primed stem cells were grown in the presence of the MEK inhibitor PD0325901, and the expression of the pluripotent marker OCT4 and the naïve-state marker H3K27me3 were measured by fluorescence microscopy. DAPI staining (blue) was used to show nuclei. The scale bars in these images represent 100 µm.

RNA-seq analysis was applied to reveal the specific genetic signature of the naïve-like PSCs. As shown in Fig. 3A, gene expression differed between naïve-like and primed PSCs. According to the RNA-seq analysis, 170 of 1043 upregulated genes were significantly upregulated [fold change > 2 (log2) with FDR < 0.01] in naïve-like cells (Supplementary Tables S1) and 92 of 737 downregulated genes were significantly downregulated [fold change < 2 (log2) with FDR < 0.01] in naïve-like cells (Supplementary Table S2). The top enriched Gene Ontology (KEGG pathway) terms for genes associated with upregulation or downregulation in naïve-like PSCs are shown in Fig. 3B. Some of the genes involved in metabolic pathways were downregulated, whereas genes involved in pluripotent and signaling pathways for MAPK, PI3K-Akt, and p53 were upregulated in naïve-like PSCs, as compared to primed PSCs. To validate the KEGG pathway analysis, the expression levels of pluripotency marker genes were determined by Q-PCR–based analysis. The pluripotency markers, OCT4 and SOX2, were significantly upregulated in naïve stem cells (Fig. 4A), even though all the exogenous viral reprogramming factors were eliminated. We also applied a pluripotency gene panel that consisted of 40 different pluripotent genes. The full list of panels for this analysis is shown in Supplementary Fig. S2. Because Otx2 is a priming factor for PSCs, its expression in naïve-like PSCs was downregulated. However, additional pluripotency genes (i.e., *NODAL*, *UTF1*, *RUNX1*, *RUNX2*, *DPPA2*, *PAX6*, *LETFY1*, and *LETFY2*) were upregulated in naïve-like PSCs (Fig. 4B).

The NODAL signaling pathway is important in embryonic development and acts as an agonist of the Smad2 signaling pathway [15]. RNA-seq determined that the mRNA expression of Smad-family genes underwent no significant change in the transition between primed and naïve-like PSCs (Supplementary Fig. S3). However, the upregulation of NODAL induced the activation of Smad2 signaling specifically in naïve-like PSCs as evidenced by increased phosphorylation of Smad2 (Fig. 4C). In contrast, Smad3 and Smad4 showed no change in their protein and phosphorylation levels in naïve-like PSCs (Supplementary Fig. S4). In addition, the ERK signaling pathway, which plays a crucial role in cellular functions (i.e., in cell proliferation, differentiation, and survival), is required for pluripotency and self-renewal in human embryonic stem cells [16]. As shown in Fig. 4C, naïve-like PSCs showed high levels of ERK2 activation. The specific inhibitor of MEK (PD0325901), which is an upstream kinase of ERK, inhibited ERK signaling in both primed and naïve-like PSCs (Fig. 4D). However, PD0325901 did not inhibit the Smad2 signaling pathway, which implies that there is ERK-independent NODAL-Smad2–axis signaling in naïve-like PSCs. Furthermore, ERK activation was involved in X-chromosome inactivation and pluripotency (Fig. 4E). As described earlier, X-chromosome inactivation in primed cells was indicated by condensed puncta after staining with an anti-H3K27me3 antibody, whereas X-chromosome reactivation in naïve-like cells resulted in cloud-like staining with an anti-H3K27me3 antibody (Fig. 4E). Treating primed and naïve-like cells with 2 μM PD3025901 resulted in the complete inactivation of ERK in both cell types. Primed cells showed the same level of punctate staining with anti-H3K27me3, whereas naïve-like cells showed cloud-like staining indicating X-chromosome reactivation, implying that X-chromosome reactivation occurs in an ERK-independent manner.

### 3.3 The reduction in ATP generation in primed PSCs is related to mitochondrial activity

**Fig. 5:**
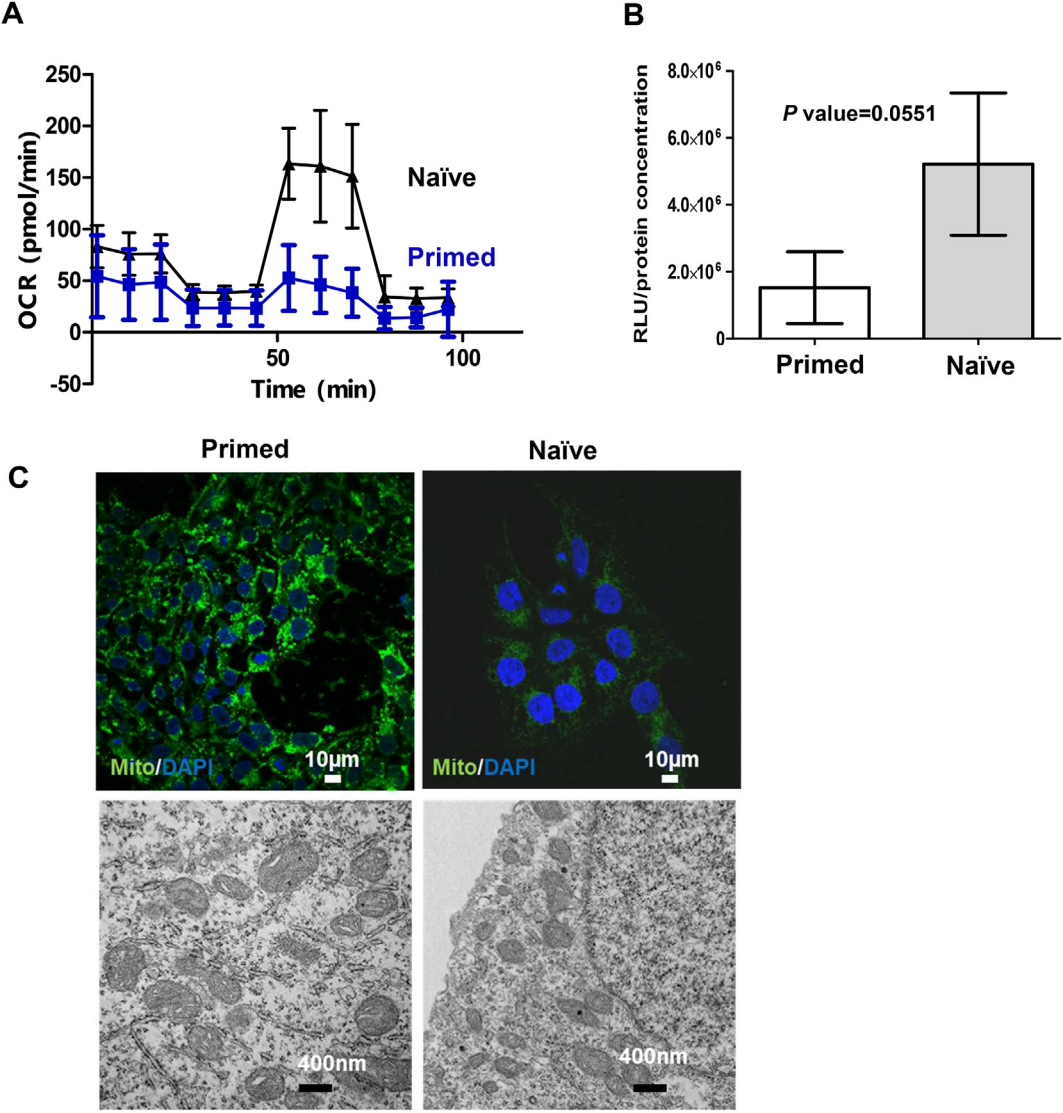
**Mitochondrial function in naïve-like stem cells converted by adding NME7AB**. **(A)** The mitochondrial oxygen consumption rate (OCR) in primed PSCs (blue squares, n = 10) and naïve-like cells (black triangles, n = 10) was measured with a Mito Stress Test Kit and a Seahorse XF24 Extracellular Flux Analyzer. The data are shown as the mean ± SD after normalization to the DNA content (in µg). **(B)** The intracellular ATP level was determined in primed and naïve-like PSCs. The measured value in RLU (relative luminescent units) was normalized to the total protein concentration. The data are shown as the mean ± SD of triplicate experiments. The statistical analysis was performed using GraphPad Prism. The results for primed and naïve-like PSCs were compared using an unpaired Student’s *t*-test (P = 0.0551). **(C)** Images of mitochondria obtained by confocal microscopy (top), using MitoTracker dye (MitoTracker Green FM) to visualize the mitochondria, and by electron microscopy (bottom). The white scale bars in the color images represent 10 µm; the black scale bars in the grayscale images represent 400 nm.

Gene Set Enrichment Analysis (GSEA) has been used as a computational method to determine significantly changed biological phenotypes and pathways in different groups by comparison with a control group for analysis. The Molecular Signatures Database (MSigDB) is one database for running GSEA. As shown in Supplementary Fig. S5, the mitochondrial pathway was upregulated in naïve-like cells, as compared to primed PSCs. In our previous study, we showed that human ESs/iPSCs exhibited decreased overall ATP production and maximal mitochondrial respiration when compared to fully differentiated fibroblasts [8]. In addition, it has been reported that a signature characteristic distinguishing naïve-like PSCs from primed PSCs is their mitochondrial function [2]. To further investigate the mitochondrial function and activity, we measured the mitochondrial oxygen consumption rate (OCR) in primed and naïve-like PSCs by using a Seahorse XF24 Extracellular Flux Analyzer. As shown in Fig. 5A, naïve-like cells (represented by black triangles) had a higher mitochondrial respiration capacity when compared to primed iPSCs (represented by blue squares). The impaired mitochondrial function in primed iPSCs could explain the reduced ATP production. As shown in Fig. 5B, the ATP production was recovered in naïve-like cells.

The suppression of mitochondrial function might result from a change in the mitochondrial number and/or mitochondrial development. This prompted us to investigate the quantity and morphology of the mitochondria present in primed and naïve-like PSCs. As shown in Fig. 5C, there was no significant difference in the number of mitochondria in primed and naïve-like cells as determined by electron microscopy and by confocal microscope examination of cells stained with MitoTracker dye. Mitochondrial dynamics play a critical role in mitochondrial function [17], and the remodeling of mitochondria formation by developing cristae is also linked to mitochondrial function [18]. In this respect, there are no quantitative differences between the mitochondria in primed PSCs and those in naïve-like PSCs. However, there are qualitative differences between these two cell populations with regard to the respiratory function as a direct result of their mitochondria.

Respiratory complexes are localized in the cristae of mitochondria. These partitions have been reported to be poorly developed in mouse and human ESCs, leading to aberrant mitochondrial OXPHOS use and defective ATP production, and this was still observed during somatic cell reprogramming [21,22]. Our electron microscopy data also supported this observation. Mitochondria in fibroblast cells had a rod-shaped morphology and fully developed cristae [23]; conversely, primed PSCs had bulging mitochondria and undeveloped cristae (Fig. 5C). Finally, mitochondria in naïve-like PSCs showed the regressive phenotype characteristic of the difference between somatic and primed PSCs, which also corresponds to known naïve-cell respiratory function.

In conclusion, these data support the hypothesis that primed iPSCs were converted to naïve-like cells by the addition of the recombinant protein NME7_AB_ and that naïve-like PSCs show full “potency of differentiation” but retain active mitochondrial function to generate more cellular energy, i.e., ATP. Major byproducts of OXPHOS are reactive oxygen species (ROS), which are notorious mutagens. Mutations in the PSCs would have a considerable impact on the cells during their development. Therefore, PSCs require the suppression of ROS generation during their development, and this could be achieved by shutting down mitochondrial OXPHOS. To compensate for this and satisfy the requirement for energy generation, PSCs have a highly active glycolysis pathway for generating ATP, although naïve PSCs also have an active mitochondrial function. Some of the critical cellular signaling pathways mediated by ROS might play a significant role in this stage of development. Further studies of the specific mechanisms of mitochondrial function in PSCs at different stages could help us to better understand and overcome some of obstacles associated with primed PSCs (i.e., lymphoid lineage differentiation).

## Conflicts of interest

The authors declare no conflict of interest.
