# Supplementary material for: The primitive growth factor NME7AB induces mitochondrially active naïve-like pluripotent stem cells
Source: Biochem Biophys Rep. 2019 Aug 20;20:100656. doi: 10.1016/j.bbrep.2019.100656 (PMC6711853; doi:10.1016/j.bbrep.2019.100656)
Supplement: Multimedia component 3 [file mmc3.docx]

**Supplementary Materials:**

Supplement Table S1. Upregulated genes in the naïve state stem cells by RNA-seq.

Supplement Table S2. Downregulated genes in the naïve state stem cells by RNA-seq.

**Supplement Figure Legends**

**Supplement Fig. 1. (A)** The primed state and naïve-like state pluripotent stem cells were differentiated to neuronal lineage: immunofluorescent assay with anti-Pax6 (Green) antibody represented differentiated neuronal progenitors. Hoechst (Blue) was used to show nucleus. **(B)** The primed state and naïve-like state pluripotent stem cells were differentiated to hematopoietic lineage: hemangioblast induction was represented by KDR ^+^ population at Day4. The hematopoietic progenitors (CD235^+^/CD41^+^) were shown in at Day8 from EB.

**Supplement Fig. 2.** The list of 40 pluripotency genes used in this study.

**Supplement Fig. 3.** The mRNA expression of Smad-family genes in primed and naïve-like PSCs was determined by RNA-seq.

**Supplement Fig. 4.** The level of phosphorylated and total Smad3/Smad4 proteins in primed and naïve-like PSCs were determined by immunoblot analysis using anti-Smad3, anti-phopho-Smad3, anti-Smad4, and anti-phospho-Smad4 antibodies.

**Supplement Fig. 5.** Gene Set Enrichment Analysis (GSEA) analysis based on MSigDB database. The mitochondrial pathway was one of upregulated pathways in naïve-like cells, as compared to primed PSCs.
