# Supplementary material for: The primitive growth factor NME7AB induces mitochondrially active naïve-like pluripotent stem cells
Source: Biochem Biophys Rep. 2019 Aug 20;20:100656. doi: 10.1016/j.bbrep.2019.100656 (PMC6711853; doi:10.1016/j.bbrep.2019.100656)
Supplement: Multimedia component 4 [file mmc4.pdf]

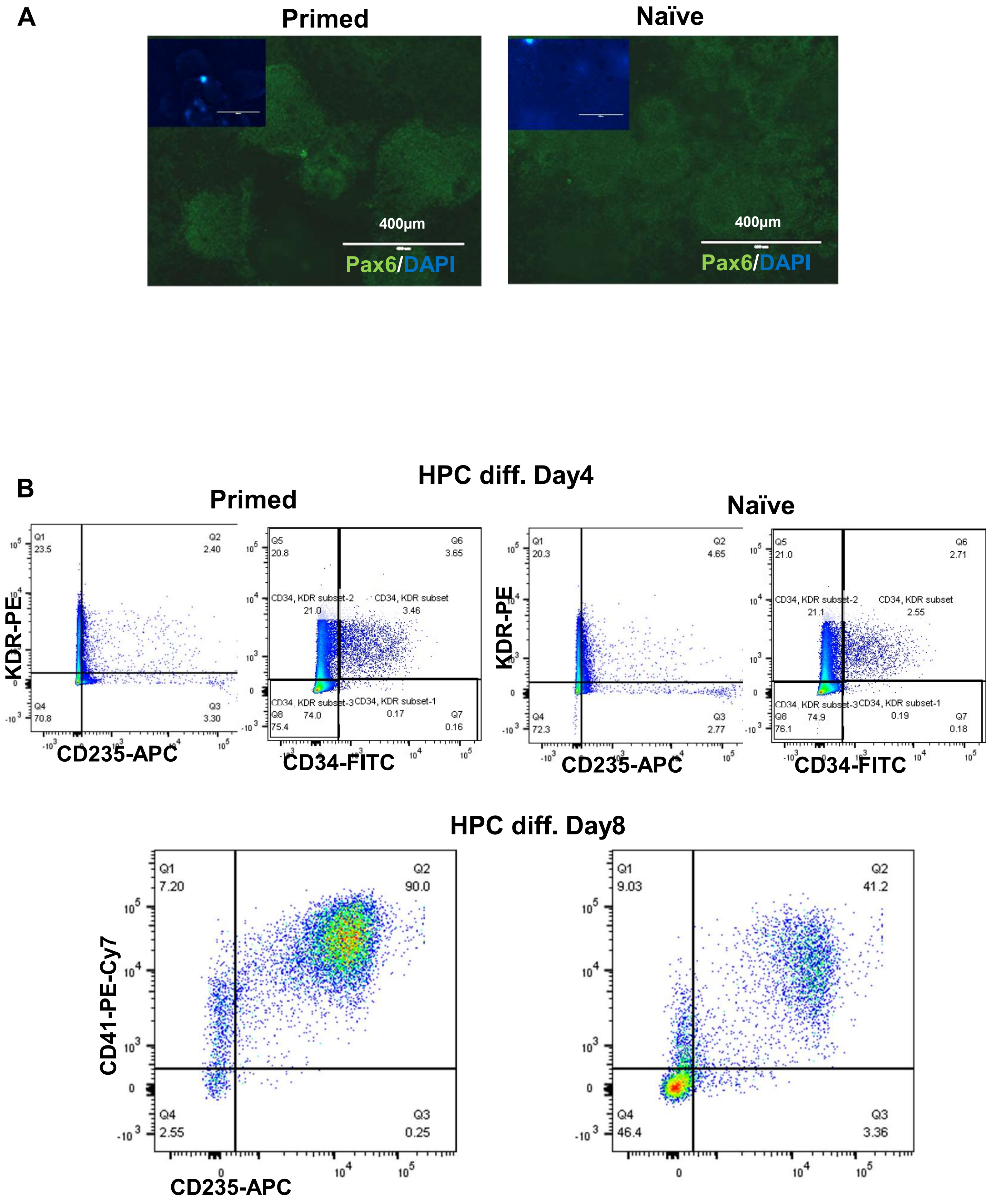

Supplement Figure S1

	1	2	3	4	5	6
A	DNMT3B	GATA2	LEFTY2	NODAL	SOX15	gDNA
B	DPPA2	GATA4	LIN28A	OLIG2	SOX17	PCR
C	DPPA3	HDAC2	MYBL2	OTX2	SOX2	RQ1
D	ESRRB	KAT2A	MYC	PAX6	TBX3	RQ2
E	FGF2	KAT7	MYCN	POU5F1	TCF3	RT
F	FGF4	KAT8	NANOG	REST	TERT	ACTB
G	FOXA2	KLF4	NAT1	RUNX1	UTF1	G6PD
H	GABRB3	LEFTY1	NCAM1	RUNX2	ZFP42	RPS18
	1	2	3	4	5	6

Supplement Figure S2

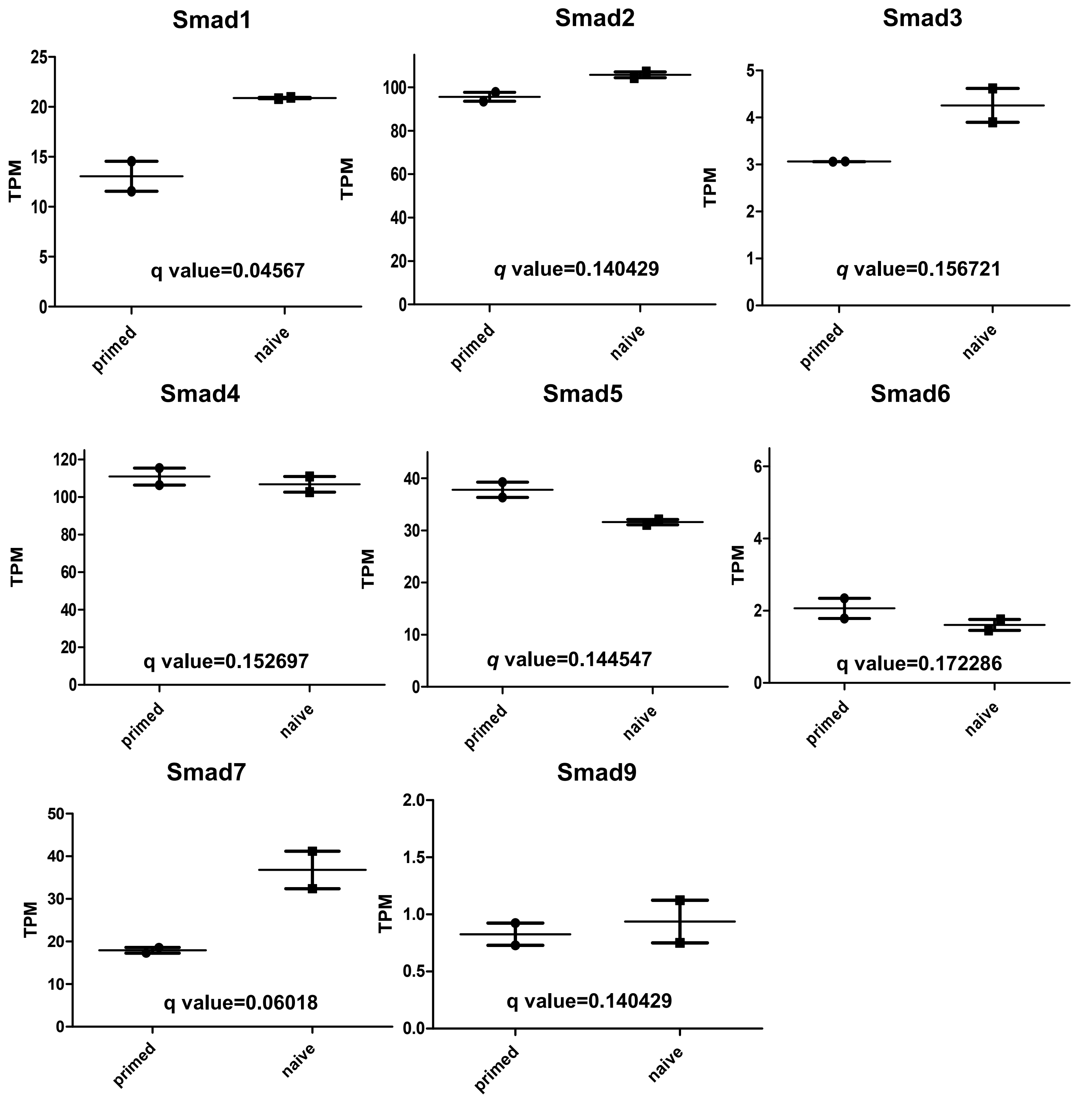

Supplement Figure S3

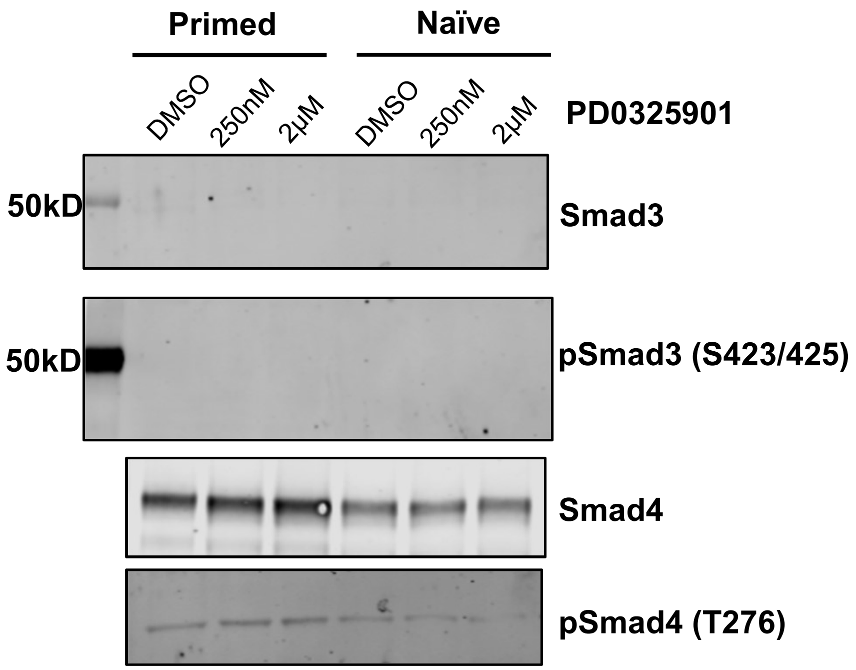

Supplement Figure S4

Up-regulated in naive

GS follow link to MSigDB	GS DETAILS	SIZE	ES	NES	NOM p-val	FDR q-val	FWER p-val	RANK AT MAX	LEADING EDGE
BIOCARTA_CDC42RAC_PATHWAY	Details ...	16	0.64	2.10	0.000	0.032	0.021	5202	tags=88%, list=30%, signal=125%
BIOCARTA_FAS_PATHWAY	Details ...	29	0.51	1.99	0.000	0.043	0.055	3450	tags=55%, list=20%, signal=69%
BIOCARTA_UCALPAIN_PATHWAY	Details ...	17	0.56	1.90	0.000	0.066	0.122	1276	tags=47%, list=7%, signal=51%
BIOCARTA_CASPASE_PATHWAY	Details ...	20	0.53	1.88	0.000	0.057	0.142	3450	tags=55%, list=20%, signal=69%
BIOCARTA_DEATH_PATHWAY	Details ...	31	0.47	1.88	0.002	0.046	0.142	3753	tags=45%, list=22%, signal=58%
BIOCARTA_INFLAM_PATHWAY	Details ...	15	0.56	1.82	0.004	0.063	0.214	3823	tags=60%, list=22%, signal=77%
BIOCARTA_MITOCHONDRIA_PATHWAY	Details ...	21	0.49	1.82	0.010	0.056	0.221	3202	tags=52%, list=19%, signal=64%

Supplement Figure S5
